# Alcohol abuse-related severe acute pancreatitis with rhabdomyolysis complications

**DOI:** 10.3892/etm.2012.735

**Published:** 2012-10-04

**Authors:** MAO-SHENG SU, YING JIANG, XIAO-YUAN HU YAN, QING-HUA ZHAO, ZHI-WEI LIU, WEN-ZHI ZHANG, LEI HE

**Affiliations:** 1 Departments of Hepatobiliary Surgery; 2 Geriatric Endocrinology and; 3 Geriatric Health Care, PLA General Hospital, Beijing 100853, P.R. China

**Keywords:** alcohol abuse, severe acute pancreatitis, rhabdomyolysis

## Abstract

Non-traumatic rhabdomyolysis is a rare complication of acute pancreatitis. One of the major risk factors of both acute pancreatitis and rhabdomyolysis is alcohol abuse. However, only a few studies have reported the prognosis and association of severe acute pancreatitis (SAP) and rhabdomyolysis in alcohol abuse patients. In the present study, we report two cases presenting with SAP complicated by rhabdomyolysis following high-dose alcohol intake. The disease onset, clinical manifestations, laboratory data, diagnosis and treatment procedure of each patient were recorded, and the association with rhabdomyolysis was analyzed. Alcohol consumption was the most predominant cause of SAP and rhabdomyolysis in these patients. SAP-related rhabdomyolysis was primarily induced by the toxicity associated with pancreatic necrosis. The laboratory tests revealed that the concentration of serum creatine kinase (CK) and myoglobin increased and acute renal failure symptoms were present, which provided an exact diagnosis for SAP-induced rhabdomyolysis. Rhabdomyolysis and subsequent hypermyoglobinuria severely impaired kidney function and aggravated hypocalcemia. The therapy of early stage SAP complicated by rhabdomyolysis involved liquid resuscitation support. When first stage treatment fails, blood purification should be performed immediately. Both patients developed multiple organ failure (MOF) and succumbed to the disease. Considering the two cases presented, we conclude that alcohol-related SAP complicated by rhabdomyolysis may have a poor clinical prognosis.

## Introduction

Acute pancreatitis is an inflammatory lesion, characterized by activation of pancreatic proenzymes with autodigestion, proteolysis, edema, vascular damage, interstitial hemorrhage, coagulation and fat necrosis. Severe acute pancreatitis (SAP) is a dangerous and complicated disease with a high mortality rate (1,2). SAP has various pathophysiological manifestations, including cardiovascular, pulmonary, renal, central nervous system and metabolic disorders (3–5). Certain cases even develop rhabdomyolysis, which is a complication rarely reported among the metabolic disorders induced by acute pancreatitis (6,7).

Rhabdomyolysis is defined as injury of skeletal muscle cells and leakage of its contents, including the enzyme creatine kinase (CK), electrolytes and myoglobin. Rhabdomyolysis is a severe pathological process caused by infection, diabetic ketoacidosis, trauma, sedation drugs, muscle relaxants and hyperthermia (8,9). Rhabdomyolysis induced by SAP belongs to the non-traumatic type.

Alcohol abuse is associated with an increased risk of acute pancreatitis and rhabdomyolysis. The pathophysiological role of alcohol in the etiology and occurrence of acute pancreatitis is complex, but increased oxidative stress, disruption of cytosolic calcium homeostasis and changes in gene expression in the pancreas appear to be involved (10,11). Ethanol also causes rhabdomyolysis via disruption of adenosine triphosphatase pump function, breakdown of the muscle membrane and alteration of the sarcoplasmic reticulum (12). However, few studies have reported the prognosis and association of SAP and rhabdomyolysis in alcohol abuse patients.

In the present study, we summarize two cases of SAP complicated by rhabdomyolysis in the Hepatobiliary Surgery Intensive Care Unit of the PLA General Hospital, Beijing, China, presenting with increased serum CK, increased myoglobin concentration and acute renal failure. We investigated characteristic clinical manifestations, laboratory findings and treatment strategy of SAP to highlight the features of this association.

## Case reports

### Case 1

#### Case history

A 30-year-old male presented with acute abdominal pain and fever for 4 days after heavy alcohol consumption and was admitted to the hospital. The patient had a history of chronic alcoholism for 10 years. The patient also had diabetes mellitus and severe fatty liver which was also observed in family members.

**Figure 1:**
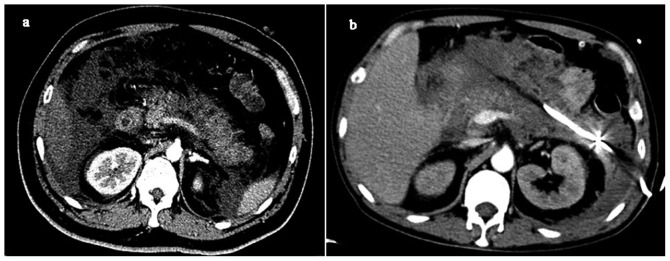
Abdominal CT scan. (a) Case 1 revealed a marked swelling and obvious pancreatic effusion. (b) Case 2 revealed a pancreatic swelling, obvious effusion and marked necrosis. CT, computed tomography.

*Physical examination.* The results of physcial examination were as follows: body temperature, 38°C; pulse rate, 160 bpm; respiratory rate, 35 bpm; and blood pressure, 110/70 mmHg. The conjunctivae were neither icteric nor anemic. The lungs were clear and heart sounds were normal. Neither hepatosplenomegaly nor edema were identified. Palpation revealed a distended abdomen and muscle tenderness on the left side. The chest roentgenogram and electrocardiogram were normal. Laboratory tests on admission indicated pancreatitis with elevated levels of serum pancreatic enzymes. Serum biochemical indicators are listed in Table I. The Ranson score at admission was 1. Ultrasonography and computed tomography (CT) of the abdomen revealed marked swelling and pancreatic effusion (Fig. 1a). An enhanced CT scan demonstrated necrosis in the pancreas and the Balthazar CT scan was grade E.

*Diagnosis.* Based on these results, we diagnosed the patient with a case of fulminating acute pancreatitis, due to alcohol intake and secondary multiple organ failure (MOF).

*Treatment strategy.* Avoidance of food and water, gastrointestinal suction, neutralization of acid, restraint of pancreatic enzyme treatment, anti-inflammatory drugs, fluid replacement and organ function support were the chosen treatment strategies for this patient. Five hours after admission, the patient developed early signs of shock that included respiratory difficulty, decreased SaO_2_ and blood pressure, and oliguria. Immediate salvage measures were performed. Intra-abdominal pressure reached 13 cmH_2_O which continued to rise. A drainage tube was inserted into the abdominal cavity to decrease the intra-abdominal pressure. On the second day after admission, patient laboratory data revealed the following: serum CK level 22,160 U/l, lactate dehydrogenase (LDH) level 4,916.2 U/l, glutamic-pyruvic transaminase (ALT) level 3,006.5 U/l and glutamic-oxalacetic transaminase (AST) level 6,578 U/l. The Ranson score at 48 h was 4 (PaO_2_ increased to 83 mmHg in 6 liters of O_2_; blood urea, 18 mmol/l; serum calcium, 1.65 mmol/l). On the third day, two additional abdominal drainage tubes were inserted. However, the abdominal pressure remained high (34 mmHg) after drainage. The effluent of blood filtration was pink. Serum potassium and myoglobin levels were elevated. CK and LDH levels were 15,180 U/l and 17,130 U/l, respectively. These parameters indicated the presence of hemolysis and rhabdomyolysis. Plasma exchange was then carried out. On the fourth day after admission, the peak CK level was 338,800 U/l; and the LDH level was 21,378.2 U/l. On the fifth day after admission, the patient’s condition markedly deteriorated, with blood pressure severely lowered and the presence of metabolic acidosis and disseminated intravascular coagulation (DIC). The patient’s family members requested to stop treatment and therapy was terminated.

### Case 2

#### Case history

A 58-year-old male was admitted to the PLA General Hospital for abdominal pain for 2 weeks and the pain was becoming more severe over 2 days. The patient had a history of heavy alcohol consumption prior to the onset of these symptoms.

*Physical examination.* Physical examination revealed the following: body temperature, 36.5°C; pulse rate, 100 bpm; respiratory rate, 30 bpm; and blood pressure, 138/87 mmHg. Abdominal distension, muscle tension and tenderness were observed. Laboratory findings on admission showed serum amylase to be 1,707.7 U/l and lipase 1,678.1 U/l (Table I). A CT scan revealed stones in the gall bladder, a pancreatic swelling, obvious effusion and marked necrosis (Fig. 1b).

*Diagnosis.* Based on these results, a diagnosis of fulminating acute pancreatitis, gall bladder lithiasis and rheumatoid arthritis was made.

*Treatment strategy.* Avoidance of food and water, gastrointestinal suctioning, neutralization of acid, control of pancreatic enzyme incretion, anti-inflammatory drugs, fluid replacement and organ function support were the selected treatment methods used. We performed endotracheal intubation and mechanical ventilation on the first day. On the second day, the patient’s laboratory data showed that the serum CK level peaked at 19,820.1 U/l; and the CK-MB level was 19.4 ng/ml. The abdominocentesis fluid was dark red blood. A drainage tube was inserted into the abdominal cavity. On the third day, the patient was treated with blood filtration, abdominal cavity pressure monitor and anti-infection treatments. Seven days after admission, the patient developed pancreatic encephalopathy. At eleven days after admission, we performed a back peritoneal incision and inserted a drainage tube. Thirteen days after admission the abdominal drainage tube presented with blood. The patient’s hemoglobin level decreased and coagulation function deteriorated. Transfusion and operation were carried out to open the abdomenal cavity for exploration and to remove the necrotic tissue around the pancreas to insert an abdominal drainage tube. After operation, the patient’s renal and coagulation function worsened, manifesting in anuria and errhysis from the incision and the abdominal drainage tube. Red blood cells (RBCs), fresh frozen plasma (FFP) and platelets were administered via transfusion. On the third day after operation, the patient had abdominal bleeding, MOF, decreased blood glucose, severe metabolic acidosis and hypotention. The patient succumbed on the fourth day after operation.

## Discussion

Severe acute pancreatitis (SAP) is a dangerous and complicated disease with a high mortality rate. The severity of SAP is assessed by the Ranson score, serious complications, organ failure and/or laparotomy (13,14). MOF presents in 93% of SAP cases (15). SAP was found to lead to a 10–30% mortality rate in the majority of prospective series (16,17). In the present study, laboratory test and CT scan results showed that serum amylase and lipase levels were increased and the pancreas displayed swelling, obvious effusion and necrosis. These clinical symptoms supported the diagnosis of SAP.

Obstruction of the common bile duct by stones (38%) and alcohol abuse (36%) are the most frequent causes of acute pancreatitis. In the present study, gall bladder lithiasis was detected by CT scan in case 2, but this symptom was not diagnosed as the main cause of SAP. Alcohol abuse is the second most frequent cause of acute pancreatitis. Acute pancreatitis develops in 10% of chronic alcohol abusers (>80 g daily intake). The present patients admitted to extreme alcohol consumption before acute abdominal pain occurred. SAP was considered the result of multiple pathogenic factors in the present cases, but alcohol abuse might be the critical direct cause. A previous study reported that alcohol plays an important role in the onset of both acute pancreatitis and rhabdomyolysis (18). In the present cases, alcohol might be contributed to both the onset and progression of SAP complicated by rhabdomyolysis.

Acute rhabdomyolysis is a complication rarely seen in acute pancreatitis. Several studies have described cases of an association between acute pancreatitis and rhabdomyolysis. Nankivell and Gillies reported, in a retrospective study, that asymptomatic rhabdomyolysis occurs in 14/548 (2.6%) of patients with acute pancreatitis (15). Another report showed that its prevalence varied from 2 to 7% (19). However, the incidence of rhabdomyolysis in SAP has not been extensively reported. In the present study, we reported two patients with severe pancreatitis developing non-traumatic rhabdomyolysis with CPK >10,000 IU/l. CK is the most sensitive indicator of muscle cell injury. There is diagnostic value for rhabdomyolysis when the serum CK level is elevated five times or more than the normal concentration. In this report, patient peak CK was elevated 3–5 days after the onset of pancreatitis and all exceeded 10,000 IU/l. Serum or urine myoglobin is another rhabdomyolysis indicator. In this report, serum myoglobin concentrations were elevated in all cases and all exceeded twice the upper normal limit. Both cases developed marked myoglobinuria. Myocardial infarction was excluded.

Pezzilli *et al* measured the serum and urine myoglobin concentrations and demonstrated a close correlation between the severity of acute pancreatitis and rhabdomyolysis (19). Pezzilli *et al* suggested that renal failure that follows acute pancreatitis is partly due to rhabdomyolysis and elevated serum concentrations of myoglobin. Rhabdomyolysis-related kidney failure is associated with a poor prognosis for patients with SAP. Hypocalcemia is another poor prognostic indicator in acute pancreatitis. When pancreatitis and rhabdomyolysis coexist, hypocalcemia is common (93%), severe and prolonged. Rhabdomyolysis may aggravate hypocalcemia. Therefore, rhabdomyolysis should be worthy of attention if patients are observed to have secondary worsening of renal function and/or profound hypocalcemia.

The treatment strategy for SAP-induced rhabdomyolysis is administration of full fluid resuscitation in order to maintain adequate urine output. Initial fluid requirements were more than 6 liters in the first 48 h. If patients still suffered oliguria after fluid resuscitation, blood purification treatment including plasma exchange or perfusion should be immediately performed. Maintaince of homeostasis and prevention of complications are also necessary.

The condition of the patients with SAP complicated by rhabdomyolysis deteriorated rapidly. Patients with a higher Ranson score and organ failure score often have a longer average intensive care unit stay, poor treatment outcome and high mortality rate. In the present cases, although standard treatment of organ support was utilized, patients were unable to recover from the severe circumstances. Intra-abdominal hypertension and rhabdomyolysis are difficult to control. All patients developed MOF and, ultimately, succumbed. A study reported that the mortality of patients with SAP and rhabdomyolysis was approximately 79% (15). The prognostic significant of rhabdomyolysis during acute pancreatitis is currently being debated. Based on the present cases, we conclude that alcohol abuse-induced SAP complicated by rhabdomyolysis demonstrated a poor prognosis and high mortality.

## Figures and Tables

**Table I:** Pathological parameters.

Indicators (units)	Case 1	Case 2
Peak CK (U/l)	338,800	19,820.1
Peak CK-MB (ng/ml)	29.6	19.4
Myoglobin (*μ*g/l)	7,315.0	2,219.0
Amylase (U/l)	1,091.9	1,707.7
Lipase (U/l)	4,093.9	1,678.1
LDH (U/l)	21,378.2	2,010.6
ALT (U/l)	3,006.5	2,114.2
AST (U/l)	14,578.1	12,046.0
BUN (mmol/l)	26.9	21.8
Cr (*μ*mol/l)	358.1	389.6
Ca (mmol/l)	1.54	1.10
P (mmol/l)	2.81	2.26
K (mmol/l)	6.3	5.5
WBCs (×10^9^/l)	20.3	15.1
Hb (g/l)	83.0	71.0
Plts (×10^9^/l)	42.0	76.0
PT (sec)	24.1	19.5
aPTT (sec)	42.8	39.3
PTA (%)	24.0	21.0
Fib (g/l)	1.93	2.25
INR	2.5	1.9

CK, creatine kinase; LDH, lactate dehydrogenase; ALT, glutamic-pyruvic transaminase; AST, glutamic-oxalacetic transaminease; BUN, blood urea nitrogen; WBCs, white blood cells; Hb, hemoglobin; Plts, platelets; PT, prothrombin time; aPTT, activated partial thromboplastin time; PTA, prothrombin activity; Fib, fibrinogen; INR, International Normalised Ratio.
